# Brain Stimulation Reward Supports More Consistent and Accurate Rodent Decision-Making than Food Reward

**DOI:** 10.1523/ENEURO.0015-17.2017

**Published:** 2017-05-01

**Authors:** Matthew S. McMurray, Sineadh M. Conway, Jamie D. Roitman

**Affiliations:** 1 Department of Psychology, Miami University, Oxford, OH 45056; 2 Department of Psychology, University of Illinois at Chicago, Chicago, IL 60607; 3 Laboratory of Integrative Neuroscience, University of Illinois at Chicago, Chicago, IL 60607

**Keywords:** Brain stimulation reward, decision, food reward, intracranial self-stimulation, preference

## Abstract

Animal models of decision-making rely on an animal’s motivation to decide and its ability to detect differences among various alternatives. Food reinforcement, although commonly used, is associated with problematic confounds, especially satiety. Here, we examined the use of brain stimulation reward (BSR) as an alternative reinforcer in rodent models of decision-making and compared it with the effectiveness of sugar pellets. The discriminability of various BSR frequencies was compared to differing numbers of sugar pellets in separate free-choice tasks. We found that BSR was more discriminable and motivated greater task engagement and more consistent preference for the larger reward. We then investigated whether rats prefer BSR of varying frequencies over sugar pellets. We found that animals showed either a clear preference for sugar reward or no preference between reward modalities, depending on the frequency of the BSR alternative and the size of the sugar reward. Overall, these results suggest that BSR is an effective reinforcer in rodent decision-making tasks, removing food-related confounds and resulting in more accurate, consistent, and reliable metrics of choice.

## Significance Statement

Food reinforcement, although commonly used, is associated with problematic confounds, especially satiety. Here, we examined the use of brain stimulation reward (BSR) as an alternative to food reward in animal models of decision-making and compared it with the effectiveness of sugar pellets. We found that BSR was more discriminable and motivated greater task engagement and more consistent preference for the larger reward in free-choice tasks. These results suggest that BSR is an effective reinforcer in rodent decision-making tasks, removing food-related confounds and resulting in more accurate, consistent, and reliable metrics of choice.

## Introduction

Food reward has been used to motivate behavior in operant tasks since the earliest works of B.F. Skinner (Skinner, 1930). Common food reinforcers include grain, sugar, saccharine, and combinations of the above. Each has strengths and weaknesses. For example, sugar pellets are highly palatable because of their sweetness, yet are also associated with a high caloric value, whereas saccharin can mimic the sweetness of sugar without postingestive effects. However, food rewards also share a common set of confounds. Food restriction is often needed to motivate high levels of engagement but also alters reward processing (Kohsaka et al., 1980; Haleem and Haider, 1996; Huether et al., 1997; Frank et al., 2005, 2012; Carr, 2007, 2011; Johnson and Kenny, 2010; Avena et al., 2013), especially in dopaminergic and serotonergic systems. Additionally, satiety may develop over the course of a behavioral session. As the animal’s desire for food declines, its motivation to optimize task performance may also decline, resulting in differential behavior and reward signaling within a single behavioral session (Aitken et al., 2016; Cone et al., 2016; West and Carelli, 2016). Using food reward also requires the experimenter to preselect reward “sizes” per trial (number of rewards or liquid concentration/volume). Thus, task performance relies on the animal’s accurate estimation of reward magnitudes, which depends on the threshold for detecting a difference (Weber’s law). This threshold is governed by sensory systems, developmental age, emotional state, and the comparison scale (Feigenson et al., 2004; Cohen Kadosh et al., 2008). Alterations to any of these neurocognitive domains could bias interpretations of otherwise intact neurophysiological systems. Last, all of these potential confounds interact, complicating attempts to replicate work (Schuck-Paim et al., 2004) and perhaps muddling our understanding of the underlying neurophysiological processes. For example, the level of food restriction and reward size combine to determine the rate of satiety development.

Depending on the task structure, the factors discussed above may have considerable effects on the accuracy and stability of the resulting behavior. In tasks relying on small numbers of trials (1–10), such as those used to assess memory, these confounds may not result in significant changes to behavior. However, studies of decision-making have historically relied on large numbers of trials, taking place over long periods of time (often multiple hours). These tasks may be more affected by the confounds associated with food rewards, depending on the time period of testing examined. Regardless, even in the case of small trial numbers, these confounds may still result in subtle changes to the neural systems involved in behavior, making translation between studies challenging.

To avoid some food-associated confounds, noncaloric reinforcers can be used, such as saccharine; however, these are rarely as motivating as food restriction and may have unforeseen behavioral consequences, such as sleep disturbances (Oishi et al., 2016) or toxicity (Whitehouse et al., 2008). Additionally, these alternatives may activate alternative sensory and reward systems (Yasoshima et al., 2015; Schier and Spector, 2016; Sclafani and Ackroff, 2016) and may still be susceptible to many of the confounds discussed above (Aoyama et al., 2014). Thus, a deeper appreciation for the confounds associated with food reward can help design more accurate experiments investigating the neurophysiological and cognitive elements of a behavior, and perhaps circumvent such confounds by using alternative approaches.

An alternative to food reward, brain stimulation reward (BSR), has been effectively used in animals models of decision-making (Rokosik and Napier, 2011; Tedford et al., 2015). Such an approach is immune to many of the confounds associated with food reward and has been shown to elicit consistent behavior that varies according to the stimulation intensity (Wise, 1996; Negus and Miller, 2014). However, we know little about the discriminability of BSR in tasks where animals freely choose between multiple rewards, the within-session consistency of BSR choice, or how BSR compares to the discriminability and stability of food rewards. Here, we systematically determined the discriminability of BSR and compared it with sugar pellet numbers in similarly structured free-choice tasks, in which animals selected the preferred reward of two options within the same reward modality. If reward magnitudes were discriminable, animals should select the “larger” of the two rewards. We additionally determined whether varying frequencies of BSR would be preferred over different numbers of sugar pellets. These results have direct implications on the use of these rewards in animal models of decision-making and will improve experimental designs in such studies, increasing the translatability of rodent findings to the human literature.

## Materials and Methods

### Subjects

Eighteen male Long-Evans rats (Charles River Laboratory) completed the study, weighing ∼300 g at the start of the study. Rats were single housed in polycarbonate cages (56 × 34 × 22 cm) and provided lab diet (LabDiet 5012) and water *ad libitum* until the completion of intracranial self-stimulation (ICSS) training (detailed below), after which animals were food-restricted to ∼90% of their free feeding weight. During food-restricted periods, animals were given access to their entire daily allotment of food ∼1.5–2 h after the completion of behavioral testing. The colony was maintained on a 12:12 light/dark cycle (7 am to 7 pm), with behavioral testing conducted in a separate experimental room during the light phase of the cycle. All procedures were conducted in accordance with the guidelines put forth by the National Institutes of Health and under the approval of the Animal Care Committee of Illinois University at Chicago.

### Surgical procedures

One week after arrival to the vivarium, each subject was anesthetized using ketamine (100 mg/kg i.p.) and xylazine (20 mg/kg i.p.), then immobilized in a stereotaxic frame. A single 11-mm bipolar stimulating electrode (PlasticsOne) was implanted unilaterally, targeting the medial forebrain bundle at the level of the lateral hypothalamus (AP: –2.8, ML: +1.7, DV: –7.8 from dura), as previously described (Carlezon and Chartoff, 2007), and was anchored to the skull via five stainless steel screws and dental acrylic. Once the surgery was complete, subjects were given Loxicom (meloxicam, 1 mg/kg s.c.) for pain relief and were allowed to recover for 7 d before testing. Once the study concluded, all subjects received a lethal dose of sodium pentobarbital (100 mg/kg i.p.) and were perfused with 10% paraformaldehyde, and brains were collected for histologic verification of electrode placement using Prussian Blue stain. Only subjects with accurate electrode placements that showed maximum rates of lever pressing for BSR of >40 presses per 50 s were included in the final dataset (four subjects removed for failure to meet lever-pressing criteria).

### Behavioral testing

Behavioral testing was conducted in standard operant chambers (Med Associates) equipped with a central pellet dispenser and two levers located on either side of a sugar pellet recepticle, with cue lights located above each lever. A speaker and house light were positioned at the rear of the chamber, and an infrared beam marked entries into the pellet recepticle. During ICSS training, each animal was connected to a flexible cable attached to a two-channel commutator (PlasticsOne) to allow for relatively free movement, and stimulation was provided via a Programmable ICSS Stimulator (Med Associates). During pellet training, animals worked for 45-mg sugar pellets (BioServ).

**Figure 1.**
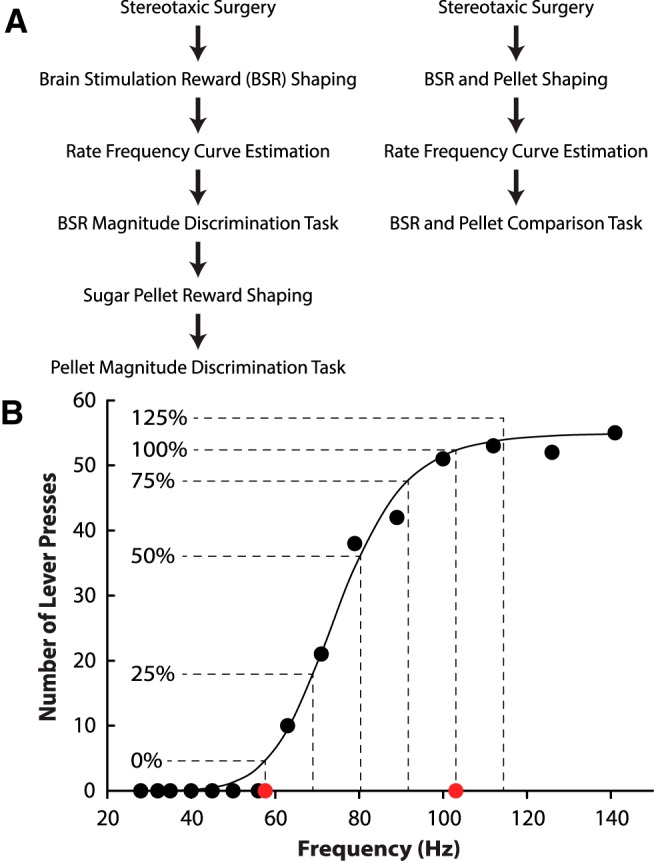
Experimental overview and sample BSR selection. ***A***, Two groups of rats were tested, 13 in the first group (left) and five in the second (right). ***B***, Sample rate–frequency curve and delineations of each reward used in the BSR tasks. Frequencies were selected for each animal based on percentages of the central linear portion of their sigmoidal rate–frequency curve.

Each stage of the experiment is detailed below and summarized in Fig. 1*A*. Thirteen animals were first trained to lever-press for brain stimulation (shaping), then individual subject rate–frequency curves were assessed to determine the full range of response-eliciting reinforcement possible for that animal. Values from this curve were used in an ICSS magnitude discrimination task. After completion of the ICSS magnitude discrimination task, animals were re-shaped to lever-press for sugar pellets, then tested in a pellet magnitude discrimination task. A second set of five animals were used in a final task, in which BSR values were compared directly with pellet reward size. This required animals to first be shaped to lever-press for both brain stimulation and pellet reward, then matched values determined across a number of sessions.

### ICSS shaping and rate–frequency curve estimation

After a 1-wk recovery from surgery, subjects were first trained to lever-press for 100-Hz brain stimulation using a fixed ratio schedule, in which each lever press earned a 500-ms train of square wave biphasic pulses. Stimulation delivery was paired with a 500-ms audible tone (4000 Hz, 100 dB). For each subject, the stimulation current was adjusted to the lowest value that sustained at least 40 responses per 50 s (final range, 60–180 μA), as in Carlezon and Chartoff (2007). Once the minimal effective current was found for each subject, it was held constant for the remainder of training and testing.

Once shaped, subjects were then trained to lever-press on a series of 15 descending stimulation frequencies (141–28 Hz, in 0.05-log10-Hz increments), each paired with one of 15 descending tone frequencies (4000–100 Hz, in 0.143-log10-Hz increments). Each frequency was available for reward during a 1-min block, which included 5 s of noncontingent stimulation (1/s), followed by 50 s of stimulation contingent on a lever press, followed by a 5-s timeout period (no stimulation was available). During the 50-s period of contingent stimulation, each stimulation was followed by a 500-ms timeout; however, all responses were counted, whether they earned stimulation or occurred during a timeout period. Subjects underwent five such passes, in which each of the 15 frequencies were presented in descending order. To determine the relationship between the rate of lever-pressing and the frequency of stimulation, sigmoidal curves were fitted to the behavioral data, and the least-squares line of best fit was plotted across the frequencies that sustained responding at 20%, 30%, 40%, 50%, and 60% of the maximum rate. The *x*-intercept of this line (Theta) was then calculated. Theta is the theoretical point at which the stimulation becomes reinforcing (Carlezon and Chartoff, 2007). Rats were trained in this manner for 3–4 wks, until Theta values became stable (±10% for three consecutive days). Once stable, animals transitioned to the BSR magnitude discrimination task.

### BSR magnitude discrimination task

To assess subjects’ ability to discriminate between stimulation magnitudes, the linear portions of their rate–frequency curves were used to establish the range of frequencies that are rewarding. Each animal’s range of frequencies between their Theta value (lowest frequency that supports pressing) and 95% of the maximum value (lowest frequency that supports maximal rates of pressing, herein referred to as Alpha) was used to calculate values for comparison in the BSR magnitude discrimination task. 95% of maximum was used because it fell more closely to the linear portion of the curve, whereas the frequency associated with the maximum rate of lever pressing often occurred in the nonlinear portion of the curve. Based on this range, the frequencies associated with Theta (0%), 25%, 50%, 75%, Alpha (100%), and 125% were then used in the BSR magnitude discrimination task (Fig. 1*B*). Thus, the frequencies and amplitudes used in the BSR task were specific to each animal’s rate–frequency curve.

Briefly, two levers were used in the BSR magnitude discrimination task, each of which was associated with one of two randomly chosen BSR frequencies (taken from each animal’s rate–frequency curve) on each session. Each session began with a block of 20 forced trials, in which only one lever was extended into the chamber at a time, and when pressed resulted in stimulation at the associated frequency. After this, a block of 100 free-choice trials followed, in which both levers were extended into the chamber, and the animal was allowed to choose the BSR frequency it preferred. Next, the reward contingencies of the levers were switched, and a second set of 20 forced-choice trials ensued, followed by a second set of 100 free-choice trials (240 trials total).

The start of each trial was signaled by the illumination of the house light. The animal was then required to make a nose-poke into the centrally located pellet receptacle to extend the levers into the chamber. A single press of either lever resulted in the immediate delivery of a 500-ms stimulation train at the associated frequency, along with a 500-ms auditory cue at the associated frequency. Immediately after the 500-ms reward, the house light was turned off, and a 5-s intertrial interval ensued, after which the house light was reilluminated to signal the availability of the next trial. Animals were given 60 min to complete all trials.

Animals first completed two sessions (one session per day) in which Alpha was assigned to both levers, followed by 22 additional sessions in which every combination of frequencies were compared twice (24 sessions total). With the exception of Alpha, no other reward frequency was compared with itself; additionally, 125% was compared only with Alpha. Behavioral performance was averaged within animal across the two repetitions of each frequency comparison.

### Pellet shaping and pellet magnitude discrimination task

After the BSR magnitude discrimination task, animals were trained to perform a similar version of the magnitude discrimination task using sugar pellet reward instead of BSR. Food-restricted animals were shaped to lever-press for a single sugar pellet using both levers on an FR1 schedule. Once shaped, animals performed 24 sessions of the pellet version of the task, during which they were asked to choose between one of two randomly chosen reward sizes (one through six pellets, 45 mg each). These sizes were chosen to match the range in reward values in the BSR task (1, Theta, 2 = 25%, etc.), and comparisons were randomized as described above in the BSR task. Within-session structure of forced-choice and free-choice trials was also matched between tasks as described above (including the postreward tone, which ranged from 4000 to 200 Hz, proportionate with reward magnitude), except that only 10 forced-choice trials and 50 free-choice trials were possible per block, to account for satiety. Animals were given 60 min to complete all trials.

Animals first completed two sessions (one session per day) in which five pellets was assigned to both levers, followed by 22 additional sessions in which every combination of pellet numbers was compared twice. With the exception of five pellets, no other reward size was compared with itself; additionally, six pellets was compared only with five pellets, mirroring the BSR task. Behavioral performance was again averaged within an animal, across the two repetitions of each reward size comparison.

### BSR and pellet comparison task

A second set of animals were food-restricted at the start of behavioral training and shaped to press one lever for brain stimulation as described above (lever side randomized between animals), followed by rate–frequency curve estimation. Once stable, animals were then shaped to press the other lever for sugar pellet reward as described above. Next, animals completed the BSR and pellet comparison task. In this task, each lever was paired with a specific BSR frequency (as described above) or number of sugar pellets with postreward tones matching those used in the two prior tasks, and the side of each association varied randomly from session to session. Each session included one set of 20 forced-choice trials, followed by 100 free-choice trials. Animals were given 60 min to complete all trials. Across 20 sessions (one session per day), comparisons were made between each BSR value (Theta, 25%, 50%, 75%, and Alpha) and two pellet reward sizes (one and two). Each comparison was made two times (two per lever side association), and performance was averaged for each animal.

### Statistical analysis

Animals were tested on each reward size comparison twice, and resulting behavior was averaged across comparable sessions, resulting in a single measure for each rat for each comparison. Thus, *n* values for each statistical comparison are equal to the number of animals tested (see above). In each comparison, preference for the larger reward was compared with chance (50%) using one-tailed *z* tests, with α levels adjusted for multiple comparisons (Bonferroni correction). Between-session preference for the larger reward was compared using one-way ANOVA, followed by *post hoc* Tukey tests when appropriate. Within-session performance was compared using two-way repeated-measures ANOVA (proportionate time × percentage difference in reward size), followed by Tukey *post hoc* tests when appropriate. Statistical analyses were completed using SigmaPlot v. 12.5 (Systat Software). All graphical results are presented as means, with error bars representing SEM. Statistical power of all statistically significant comparisons exceeded 0.85, and no outliers were excluded from any statistical comparison. All statistical methods are presented in Table 1.

**Table 1.** Statistical tests and values

Graph	Type of test	Statistical values
a. Fig. 2A, preference for larger reward	*z* tests (50% preference; one-tailed); ANOVA (% difference in compared BSR frequencies)	*p* < 0.001 (25–100% difference in BSR)*F_(3,45)_ = 13.1, *p* < 0.001*
b. Fig. 2B, preference for larger reward	*z* tests (50% preference; one-tailed); ANOVA (individual frequency comparisons)	*p* < 0.05 (all BSR comparisons)*F_(10,118)_ = 6.995, *p* < 0.001*
c. Fig. 2C, preference for larger reward	*z* tests (50% preference; one-tailed); ANOVA (raw difference in compared BSR frequencies)	All *p* < 0.001 (all BSR comparisons)*F_(5,37)_ = 3.61, *p* = 0.011*
d. Fig. 2D, number of trials completed	ANOVA (individual frequency comparisons)	*F*_(10,118)_ = 2.446, *p* = 0.011*
e. Fig. 2E, preference for larger reward	*z* tests (50% preference; one-tailed); ANOVA (proportionate time × % difference in BSR frequency); ANOVA (proportionate time); ANOVA (% difference in BSR frequency)	*p* ≤ 0.01 (all BSR comparisons)*F_(27,279)_ = 1.346, *p* = 0.133*F*_(9,279)_ = 5.838, *p* < 0.001*F_(3,279)_ = 20.582, *p* < 0.001*
f. Fig. 2F, number of trials completed	ANOVA (proportionate time × % difference in BSR frequency); ANOVA (proportionate time); ANOVA (% difference in BSR frequency)	*F*_(27,279)_ = 1.242, *p* = 0.205*F*_(9,279)_ = 6.320, *p* < 0.001*F_(3,279)_ = 1.457, *p* = 0.259
g. Fig. 3A, preference for larger reward	*z* tests (50% preference; one-tailed); ANOVA (difference in pellet number)	*p* < 0.001 (all pellet comparisons)*F_(3,39)_ = 8.63, *p* < 0.001*
h. Fig. 3B, preference for larger reward	*z* tests (50% preference; one-tailed); ANOVA (individual pellet comparisons within each comparison range)	*p* < 0.001 (1v2, 2v3, 1v3, 2v4, 1v4, 2v5, 1v5)*F_(10,118)_ = 7.00, *p* < 0.001*
i. Fig. 3C, number of trials completed	ANOVA (individual pellet comparisons within each comparison range)	*F*_(10,109)_ = 5.40, *p* < 0.001*
j. Fig. 3D, preference for larger reward	Pearson correlation	*R* ^2^ = 0.998, *p* < 0.0001*
k. Fig. 3E, preference for larger reward	*z* tests (50% preference; one-tailed); ANOVA (proportionate time × % difference in pellet number); ANOVA (proportionate time); ANOVA (% difference in pellet number)	*p* ≤ 0.05 (0.0–0.1 proportionate time)*F_(27,399)_ = 1.509, *p* = 0.056*F*_(9,399)_ = 25.29, *p* < 0.001*F_(3,399)_ = 1.91, *p* = 0.152
l. Fig. 3F, number of trials completed	ANOVA (proportionate time × difference in pellet number); ANOVA (proportionate time); ANOVA (% difference in pellet number)	*F*_(27,399)_ = 0.878, *p* = 0.64*F*_(9,399)_ = 112.136, *p* < 0.001*F_(3,399)_ = 1.146, *p* = 0.349
m. Fig. 4A, preference for 1 sugar pellet	*z* test (50% preference; one-tailed); ANOVA (proportionate BSR frequency)	*p* < 0.05 (0%, 50% BSR)*F_(4,24)_ = 0.413, *p* = 0.80
n. Fig. 4B, preference for 2 sugar pellets	*z* test (50% preference; one-tailed); ANOVA (proportionate BSR frequency)	*p* < 0.001 (0%, 25% BSR)*F(_4,24)_ = 0.963, *p* = 0.449
o. Total pellets earned	ANOVA (sugar pellet reward size × BSR reward size); ANOVA (sugar pellet reward size); ANOVA (BSR reward size)	*F*_(4,49)_ = 1.486, *p* = 0.253*F*_(1,49)_ = 66.31, *p* < 0.001*F_(4,49)_ = 1.037, *p* = 0.418
*p*. Fig. 4C, preference for 1 sugar pellet	*z* test (50% preference; one-tailed); ANOVA (proportionate time × proportionate BSR frequency); ANOVA (proportionate time); ANOVA (proportionate BSR frequency)	All *p* < 0.05 (0.3–1.0 proportionate time)*F_(36,249)_ = 0.928, *p* = 0.59*F*_(9,249)_ = 3.987, *p* = 0.001*F*_(4,249)_ = 0.82, *p* = 0.531
q. Fig. 4D, preference for 2 sugar pellets	*z* test (50% preference; one-tailed); ANOVA (proportionate time × proportionate BSR frequency); ANOVA (proportionate time); ANOVA (proportionate BSR frequency)	All *p* < 0.05 (0.6–1.0 proportionate time)*F_(36,249)_ = 1.886, *p* = 0.005*F_(9,249)_ = 10.60, *p* < 0.001*F_(4,249)_ = 1.295, *p* = 0.314
r. Fig. 4E, number of trials completed (1-pellet sessions)	ANOVA (proportionate time × proportionate BSR frequency); ANOVA (proportionate time); ANOVA (proportionate BSR frequency)	*F*_(36,249)_ = 0.916, *p* = 0.608*F*_(9,249)_ = 3.353, *p* = 0.004*F_(4,249)_ = 1.538, *p* = 0.239
s. Fig. 4F, number of trials completed (2-pellet sessions)	ANOVA (proportionate time × proportionate BSR frequency); ANOVA (proportionate time); ANOVA (proportionate BSR frequency)	*F*_(36,249)_ = 1.360, *p* = 0.105*F*_(9,249)_ = 4.673, *p* < 0.001*F_(4,249)_ = 0.606, *p* = 0.664

## Results

### BSR magnitude discrimination

**Figure 2.**
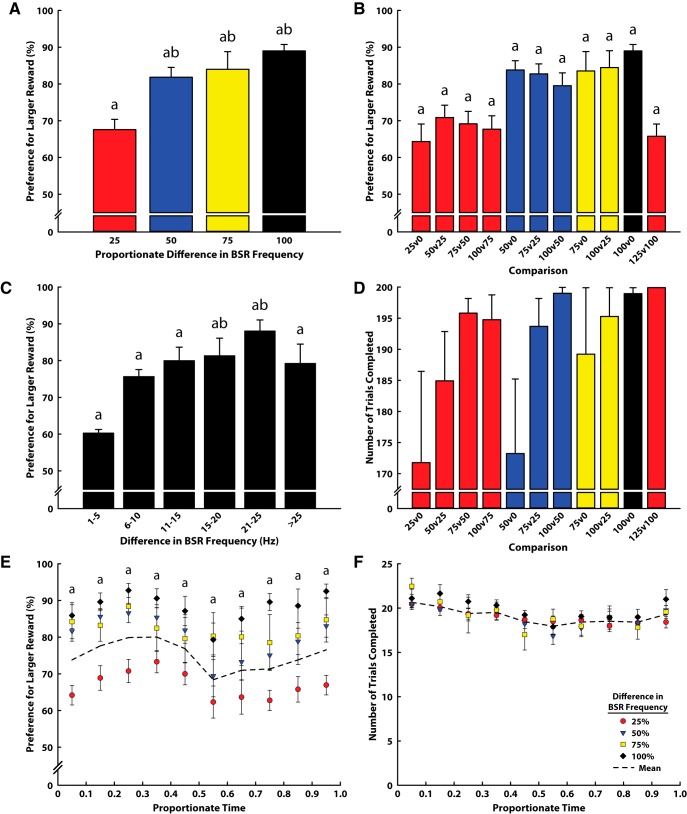
Results from the BSR magnitude discrimination task. In all panels, a indicates significant difference from chance responding (50% preference, *p* < 0.001). ***A***, Relationship between the proportionate difference in reward size and the animals’ preference for the larger reward. b indicates significant difference from preference at 25% difference (*p* < 0.01). ***B***, Preference for the larger reward at each possible reward comparison. Statistical comparisons were made only within comparison groups (e.g. within 25% difference). ***C***, Effect of raw frequency difference in BSR values on preference for the larger reward. b indicates significant difference from preference at 1- to 5-Hz difference (*p* < 0.01). ***D***, Number of trials completed in each comparison. There was no effect of comparison on the number of trials completed. ***E***, Preference for the larger reward over the course of the average session (time normalized across sessions), at each level of proportionate difference in reward size. Dotted line illustrates the mean of all comparisons, and significance is denoted only for this mean. There was no effect of time on preference. ***F***, Trial completion rate over the course of the average session (time normalized across sessions), at each level of proportionate difference in reward size. Dotted line illustrates the mean of all comparisons. There was no effect of time on preference.

To assess the discriminability of various frequencies of stimulation of the lateral hypothalamus/medial forebrain bundle, we allowed animals to select their preferred stimulation in a free-choice task. Each animal was first shaped to lever-press for stimulation, and rate–frequency curves then generated. Once stable, six reward values spanning the linear range of each animal’s rate–frequency curves were computed for comparison in the discrimination tasks (Fig. 1). From the linear portion of each animal’s curve, Theta (0%) was calculated as the minimally reinforcing frequency, and Alpha (100%) as the frequency supporting 95% maximal responding, with three steps in between (25%, 50%, 75%), and one supermaximal frequency (125%). In each session, animals were given the choice between a pair of BSR frequencies, with one higher than the other. Comparisons of randomized pairs of frequencies were made over successive days o*f* testing, with the expectation that highly discriminable stimuli would result in a clear preference for one frequency over the alternative. Within this experimental context, we examined how the percentage difference and raw difference in the stimulation frequencies dictated animals’ choices and the number of trials each animal completed. We found that proportionately larger rewards (percentage difference) were always preferred above chance (Fig. 2*A*; *z* tests; all *p* < 0.001). As the percentage difference of compared frequencies increased, animals’ choices of the higher frequency became more predominant (Fig. 2*A*; one-way ANOVA; *F*_(3,45)_ = 13.1, *p* < 0.001). Specifically, frequencies differing by >25% of the animals’ range all differed significantly from 25% difference in frequencies (Tukey *post hoc*; all *p* ≤ 0.01). Although the magnitude of frequency difference affected their discriminability, the exact position within the range of rewarding values did not (for example, 0% vs. 25%, compared with 75% vs. 100%, both of which differ by 25%). Within each comparison range (i.e., 25% difference), comparisons at the low end of the range were just as discriminable as those at the high end of the range (within percentage, ns; Fig. 2*B*), even when including a value that exceeded the linear portion of the animal’s rate–frequency curve. Specifically, 125% versus 100% was no more discriminable than any other 25% difference, suggesting that discriminability may not be restricted to the linear portion of the rate–frequency curve.

We also examined how the raw difference between frequencies controlled the discriminability of each comparison. We found that larger rewards were always preferred above chance, regardless of the raw difference between frequencies compared (Fig. 2*C*; *z* tests; all *p* < 0.001). Additionally, a one-way ANOVA found that there was an effect of frequency (*F*_(5,37)_ = 3.61, *p* = 0.011), with Tukey *post hoc* tests demonstrating that frequencies differing by 15–20 and 21–25 Hz resulted in stronger preferences for the larger reward than frequencies differing by only 1–5 Hz (Fig. 2*C*; both *p* < 0.01). However, frequency comparisons differing by 6 Hz or more did not differ from each other.

Next, we examined how each comparison dictated the number of trials animals completed. Animals could voluntarily complete up to 200 free-choice trials in each hour-long session. In all sessions, the average trial completion rate exceeded 85% of available choice trials, indicating a high level of task engagement. Additionally, we found that animals completed fewer trials when the frequencies compared were both in the lower 50% of the animals’ rewarding range (Fig. 2*D*), although this relationship was not statistically significant when corrected for multiple comparisons. Animals approached 200 trials when one value included the animal’s Alpha, and all animals completed 200 trials when both stimulation frequencies were at the animals’ Alpha or above.

Last, to determine whether satiety, exhaustion, or other behavioral biases influenced preference for the larger reward, we examined how choice changed over the course of the average session for each comparison. Because the rate of lever-pressing differed between animals, we normalized time across sessions, including only the time during free-choice trials, and compared the number and proportion of presses that occurred in time bins of 10% the total free-choice session time. We found that regardless of the comparison, preference for the larger reward was stable over the entirety of the session (see Fig. 2*E*), and animals completed the session at approximately the same pace (see Fig. 2*F*). In many of the comparisons, there was a small decrease in preference for the larger reward during the sixth bin (0.5–0.6), but this decrease was not statistically significant. Additionally, preference for larger rewards remained stably above chance level at all time bins for all comparisons (*z* tests; all *p* ≤ 0.01).

### Sugar pellet magnitude discrimination

**Figure 3.**
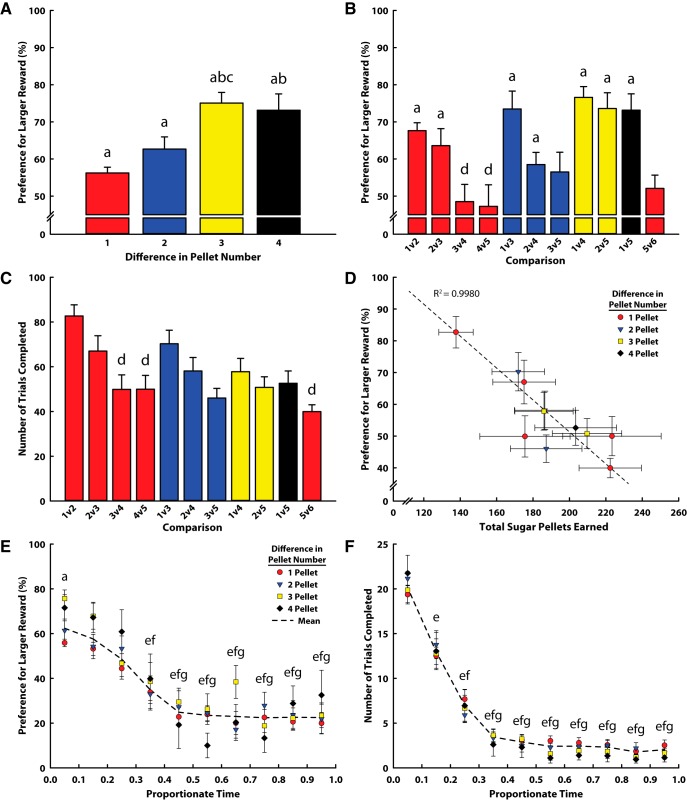
Results from the sugar pellet magnitude discrimination task. In all panels, a indicates significant difference from chance responding (50% preference, *p* < 0.001). ***A***, Relationship between the difference in reward size (pellet number) and the animals’ preference for the larger reward. b indicates significant difference from one pellet (*p* < 0.01); c indicates significant difference from two pellets (*p* < 0.05). ***B***, Preference for the larger reward at each possible reward comparison. Statistical comparisons were only made within comparison groups (e.g., within one-pellet difference). d indicates significant difference from one-versus-two comparison (*p* < 0.05). ***C***, Number of trials completed in each comparison. Statistical comparisons were only made within comparison groups (e.g., within one-pellet difference). d indicates significant difference from one-versus-two comparison (*p* < 0.01). ***D***, Relationship between the average total number of sugar pellets earned in each comparison and the animals’ preference for the larger reward. Dotted line denotes significant linear relationship between these values (*R* ^2^ = 0.998, *p* < 0.0001). ***E***, Preference for the larger reward over the course of the average session (time normalized across sessions), at each difference in reward size. Dotted line illustrates the mean of all comparisons, and significance is denoted only for this mean (there was no significant effect of comparison). e, f, and g denote significant difference from bins 0–0.1, 0.1–0.2, and 0.2–0.3, respectively (all *p* < 0.001). ***F***, Trial completion rate over the course of the average session (time normalized across sessions), at each level of proportionate difference in reward size. Dotted line illustrates the mean of all comparisons, and significance is denoted only for this mean (there was no significant effect of comparison). e, f, and g denote significant difference from bins 0–0.1, 0.1–0.2, and 0.2–0.3, respectively (all *p* < 0.001).

To compare BSR discriminability to the discriminability of rewards commonly used to reinforce performance in rodent behavioral tasks (sugar pellets ranging in number, one to six pellets), we allowed the same animals to select their preferred number of pellets in a free-choice task similar to the task used to assess their preferred BSR. In each session, animals were given the choice between a larger or smaller number of sugar pellets. Comparisons of various numbers of pellets were made over successive days of testing. Highly discriminable differences in the number of sugar pellets should result in a clear preference for the larger number of pellets, since animals are food restricted and thus motivated to perform the task as efficiently as possible. We therefore examined how differences in pellet number influenced animals’ choices and the number of trials each animal completed. We found that reward size differences as small as one pellet resulted in preferences for the larger reward greater than chance (Fig. 3*A*; *z* tests; all *p* < 0.001). In general, animals’ preference for the larger number of pellets increased with the difference in pellet number (Fig. 3*A*; one-way ANOVA; *F*_(3,39)_ = 8.63, *p* < 0.001). Specifically, comparisons differing by only one pellet showed reduced discriminability compared with those differing by three and four pellets (Tukey *post hoc*; both *p* < 0.01), whereas comparisons differing by three pellets also resulted in greater discriminability than those differing by only two pellets (*p* < 0.05). However, when individual comparisons were examined separately (Fig. 3*B*; one-way ANOVA; *F*_(10,118)_ = 7.00, *p* < 0.001), we found that the apparent reduction in discriminability when rewards differed by only one pellet was due to sessions in which larger numbers of pellets were compared (three vs. four and four vs. five). Tukey *post hoc* tests showed that one versus two pellets was considerably more discriminable than three versus four (*p* = 0.045) and four versus five (*p* = 0.024) pellets. While not statistically significant, this same trend can be observed in two-pellet comparisons (one vs. three, two vs. four, three vs. five). Additionally, only a few comparisons resulted in preferences above chance (statistically different from 50% preference). These included one versus two, two versus three, one versus three, one versus four, two versus five, and one versus five (*z* tests; all *p* < 0.001). The remaining comparisons did not result in significant discriminability.

We next examined how engagement in the task depended on the number of sugar pellets compared. As a gross measure, we first looked at the number of trials completed during each session (100-trial maximum), and saw significant variation across comparisons (Fig. 3*C*; one-way ANOVA; *F*_(10,109)_ = 5.40, *p* < 0.001). This relationship was most prominent (and significantly different) in the comparisons of only one-pellet difference, where three versus four (Tukey *post hoc*; *p* < 0.001), four versus five (*p* = 0.002), and five versus six (*p* < 0.001) were all significantly lower than one versus two. This trend persisted across all comparisons; animals completed fewer trials in sessions in which individual trials resulted in delivery of more pellets (Fig. 3*D*; Pearson correlation; *R* ^2^ = 0.998, *p* < 0.0001), suggesting that engagement in the task may have depended on the satiety of the animal. Overall, the total number of trials completed fell well short of the completion rate observed for BSR (Fig. 2*D*).

To investigate the role of satiety further, we looked at average within-session behavioral patterns. We saw that animals’ preference for the larger option decreased over the 1-h session (Fig. 3*E*) and that they completed trials at a decreasing rate as time elapsed (Fig. 3*F*). For measurements of preference and rate of trial completion, there were no noteworthy differences between comparisons (all pellet comparisons were approximately the same); however, there was a significant effect of time on both the preference for the larger reward (repeated-measures two-way ANOVA; *F*_(9,399)_ = 25.29, *p* < 0.001) and the number of trials completed per bin (repeated-measures two-way ANOVA; *F*_(9,399)_ = 112.136, *p* < 0.001). Compared with the first and second time bins (0–0.1 and 0.1–0.2), preference for the larger reward was significantly lower in all bins after 0.3 (Tukey *post hoc*; all *p* < 0.001), and compared with the third time bin (0.2–0.3), preference was significantly decreased in all bins after 0.4 (all *p* < 0.001). Additionally, only the first time bin consistently demonstrated preferences above chance (*z* test; *p* ≤ 0.05), with the exception of two-pellet comparisons, which did not reach statistical significance at any time bin. The number of trials completed in each time bin followed a similar pattern, showing significantly decreased trial numbers in the later bins of the task. Time bin 0–0.1 was significantly higher than all other bins (Tukey *post hoc*; all *p* < 0.001), whereas time bin 0.1–0.2 differed from all later time bins (all *p* < 0.001), and time bin 0.2–0.3 also differed from all later bins (all *p* < 0.001). After time 0.3, the number of trials animals completed remained stable, but very low.

### BSR and sugar pellet comparison

**Figure 4.**
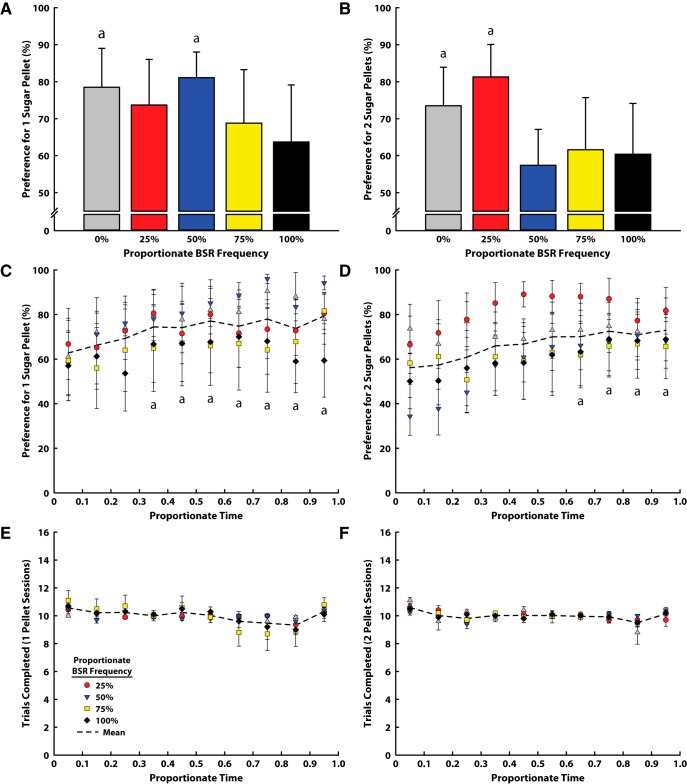
Results from the BSR and sugar pellet comparison task. In all panels, a indicates significant difference from chance responding (50% preference, *p* < 0.001). ***A***, Preference for one sugar pellet over BSR reward. There was no effect of BSR reward size. ***B***, Preference for two sugar pellets over BSR reward. There was no effect of BSR reward size. ***C***, Preference for one sugar pellet over the course of the average session (time normalized across sessions), at BSR alternative. Dotted line illustrates the mean of all comparisons, and significance is denoted only for this mean (there was no significant effect of comparison). ***D***, Preference for two sugar pellets over the course of the average session (time normalized across sessions), at each BSR alternative. Dotted line illustrates the mean of all comparisons, and significance is denoted only for this mean (there was no significant effect of comparison). ***E***, Trial completion rate in the one-pellet version of the task over the course of the average session (time normalized across sessions), at each BSR alternative. Dotted line illustrates the mean of all comparisons, and significance is denoted only for this mean (there was no significant effect of comparison). ***F***, Trial completion rate in the two-pellet version of the task over the course of the average session (time normalized across sessions), at each BSR alternative. Dotted line illustrates the mean of all comparisons, and significance is denoted only for this mean (there was no significant effect of comparison).

In a final experiment, we determined whether animals preferred BSR or sugar pellet reward in a similar free-choice task. In a task modeled after those used above, food-restricted animals were given the choice between sugar pellet reward (one or two available in each session) and BSR (one value in each session, selected from each animal’s rate–frequency curve, as discussed above). When asked to choose between one sugar pellet and BSR, animals’ preference for one sugar pellet did not depend on the stimulation frequency of the alternative reward (one-way ANOVA; Fig. 4*A*; *F*_(4,24)_ = 0.413, *p* = 0.80). Furthermore, one-pellet preference exceeded chance when compared with 0% and 50% BSR (*z* test; both *p* < 0.05). When asked to choose between two sugar pellets and BSR, a similar result was found. Animals’ preference for two sugar pellets did not depend on the stimulation frequency of the alternative reward (Fig. 4*B*), although there was a nonsignificant decrease in preference for two sugar pellets when animals could choose higher-frequency BSR (one-way ANOVA; *F*_(4,24)_ = 0.963, *p* = 0.449). Additionally, two-pellet preference exceeded chance when compared with 0% and 25% (*z* test; both *p* < 0.001). Although animals earned significantly more sugar pellets in the two-pellet comparisons (one pellet, 73.2 ± 5.3; two pellets, 133.7 ± 5.3; one-way ANOVA; *F*_(1,49)_ = 66.31, *p* < 0.001), behavioral data did not support the idea that animals chose pellets early in the task, and once sated, shifted to BSR (Figs. 4*C*, *D*). Rather, there was a trend toward increased preference for pellets over time, which exceeded chance only after the third bin in the one-pellet comparison (*z* test; all *p* ≤ 0.05) and after the sixth bin in the two-pellet comparison (*z* test; all *p* ≤ 0.05). Additionally, all animals completed all 100 trials allowed in the 1-h test period, and we found no difference in the number of trials animals completed over time (Figs. 4*E*, *F*), suggesting that animals were highly engaged while performing the task.

## Discussion

In light of the confounds associated with the use of food reward in rodent tasks of decision-making and reward, we explored the use of BSR as an alternative reinforcer. To validate its use and determine whether BSR supports reliable choice patterns, we compared the discriminability of differing numbers of sugar pellet rewards to differing frequencies of brain stimulation reward. We found that discrimination of BSR was much more robust, accurate, and consistent than that of sugar pellet reward. Animals reliably detected discrepancies in BSR frequencies differing by even small amounts (<5 Hz), although larger discrepancies between rewards resulted in stronger preferences. This did not depend on the specific frequencies used, but rather the proportionate difference between them. The was almost no variation in preference over the course of a session, and animals showed very high levels of engagement regardless of frequencies compared (as measured by the number of trials animals completed). In comparison, sugar pellet reward elicited comparatively weaker preferences for larger rewards, which greatly depended on the scale of rewards compared, changed dramatically over the course of a session, and elicited much lower levels of task engagement. Combined, these results agree with previous studies demonstrating that BSR is an effective reinforcer in rodent decision-making tasks (Rokosik and Napier, 2011; Tedford et al., 2015; Holtz et al., 2016), and extend these studies to demonstrate that BSR may result in more reliable metrics of choice than sugar pellets.

Previous research on BSR discriminability typically made only one frequency of stimulation available at any given time in the session and assessed the number of lever presses animals made for that frequency. In this manner, animals were sequentially exposed to a number of frequencies, and rate–frequency curves were generated for each animal. Although this method of study assesses the animal’s motivation to work for the higher-frequency stimulation, it fails to address which reinforcers are actually preferred by the animal, which is a common outcome of many decision-making tasks. Recent decision-making research with BSR demonstrated that animals do have clear preferences for higher-frequency stimulations (Rokosik and Napier, 2011; Tedford et al., 2015) but did not explore the full range and limitations of these preferences. Here, we have defined clear upper and lower boundaries to these preferences. Our data suggest that future studies should use frequencies that differ by at least 5 Hz, although greater differences in frequency are associated with greater discriminability in behavioral results. Additionally, to maximize the number of trials animals completed, at least one frequency should be chosen from the upper three-quarters of the animal’s rate–frequency curve. Using these parameters, highly accurate assessments of decision-making are possible, even when using very small differences in reward size. Using these methods will allow for multiple reward sizes to be accurately generated for each animal, facilitating a richer understanding of decision-making and its underlying neurocircuitry.

Our results on sugar pellet number discrimination are in good agreement with previous findings presented on the topic. In a similar task, rats’ choices for a larger number of pellets decreased as the ratio between the reward sizes became smaller (Cox and Montrose, 2016). Our data agree with this, and furthermore indicate that good discriminability of one-pellet differences exists only when the maximal reward size is low (two to three pellets). Similarly, the discriminability of two-pellet differences become less clear when maximal reward size exceeds three pellets, whereas differences larger than two pellets seem to elicit clear preferences for the larger reward within the ranges tested here. However, as the overall magnitude of pellets increases beyond those studies here (e.g., six vs. eight or eight vs. 10), a difference of two pellets is likely to be less discriminable and satiety likely achieved more quickly (see Fig. 2*C*). Thus, the data presented here support the conclusion that rats approximate pellet numbers when performing quantity discriminations, much like human infants when assessed in a similar task (Feigenson et al., 2002). However, it should be noted that from the data presented here, we cannot determine whether comparisons that showed no clear preference were indiscriminable or equally valued, since both options could have resulted in a lack of preference. Thus, these results do not address potential perceptual differences between reward sizes and should not be interpreted as an inability to perceive differences in reward size, but instead simply a preference or lack thereof.

The data from the sugar pellet task also highlights the importance of total reward size in task design. As the number of pellets earned per trial increased, the number of trials animals completed in a session decreased, as did the choice of the larger reward. This result may have strong implications on study designs relying on large numbers or trials, such as those commonly used in animal models of decision-making. Thus, researchers wishing to obtain clear choice outcomes while maximizing the number of trials animals complete should balance total reward size with the magnitude of difference in the comparison. In our opinion, the choice between one and three sugar pellets (45 mg) best achieves this optimal balance.

Of particular note was the decline in preference for the larger sugar reward over the course of a session, regardless of reward size or comparison. This is highly concerning, considering that many researchers typically average behavioral performance over the course of a session. This decline implies that in behavioral studies using food reward, the neurocognitive systems engaged early in a session may differ dramatically from those used later in a session, potentially confounding countless studies of decision-making. The rate of this decline is especially concerning, suggesting that a significant number of trials may be affected by this serious confound, and manipulations that reduce engagement early in the session may complicate this issue even more. Whether this warrants reinvestigation of previous findings is not clear; however, it does suggest that future research using sugar pellet reward should examine more than simply the average of choices over the course of a session. It is especially worth noting that the steep decline in task engagement in the pellet discrimination task was not observed when animals chose between pellets and BSR. When both reward modalities were available, animals maintained greater levels of participation, despite fewer pellets obtained overall. This supports the idea that reducing the rate of pellets earned over the course of a session can aid in maintaining task engagement.

One curious finding reported here is that animals did not show strong preference for BSR over sugar pellets, even when animals were rewarded with only a single pellet. This contradicts common knowledge of BSR, that animals will “self-starve” in favor of stimulation (Stutz et al., 1971; Santos and Routtenberg, 1972), but may be reflective of variations in electrode placement. More medial placements may induce this effect, and more lateral placements may escape self-starvation (Barbano et al., 2016; Gigante et al., 2016). Given the relative homogeneity of our results, in terms of both choice of sugar pellet and electrode location, it is difficult to determine whether our placements could have dictated this result. Additionally, much of the older literature on self-starvation relied on Rat Chow pellets, as opposed to sugar pellets, which likely differentially activate the reward circuitry due to different palatability, perhaps driving greater preference of food reward. Indeed, rats have been shown to prefer sweet rewards over some drugs of abuse (Madsen and Ahmed, 2015), supporting this hypothesis. Last, the severity and timing of food restriction may also play a role in animals’ preference for sugar (Frank et al., 1981; Burnett et al., 2016). Clearly, more research into the complexities of this result are warranted; however, in light of these findings, perhaps a tempering of the assumption that BSR is more rewarding than food is justified.

The results from this set of experiments suggest that BSR can be used to motivate more accurate, consistent, and reliable decision-making than sugar pellet rewards and suggest the use of specific reinforcer magnitudes to optimize task performance, for both pellet-based and BSR-based tasks. It should be noted that like pellet reward, BSR is not impervious to manipulation, as evidenced by its use in rate–frequency curve-shift studies (Carlezon and Chartoff, 2007). However, BSR values in choice tasks could be modified to account for shifted curves, reducing the potential impact of any confound. Such an improvement would be difficult to accomplish in food-rewarded tasks, but is easily accomplished using BSR-based tasks. Additionally, it should be noted that the order of testing was not counterbalanced in our design. Counterbalancing would have led to differential histories of food restriction, potential confounding results as discussed above. Future studies may wish to explore any potential impact of this factor. Regardless, the results presented here suggest a major improvement to future studies of rodent decision-making, increasing the translatability of findings to human studies that rely on monetary reward, and improving the replicability of findings within the animal literature.

Although these results demonstrate that BSR has clear advantages over sugar pellets, BSR also introduces a number of experimental factors that must be considered before adopting, many of which could affect results independently of other factors. First, before BSR can be used, animals must undergo a stereotaxic surgery to implant the stimulating electrode. The use of a surgical procedure necessitates additional protocols to ensure the welfare of the animal and is typically associated with a monetary cost to the investigator (supplies, access to surgical facility, etc.). Electrode misplacement during surgery results in the removal of the subject from study, lowering statistical power or requiring additional animals be included. In our hands, study sizes need to be ∼10% larger to accommodate this potential attrition. Additionally, to maintain electrode patency, our animals were single-housed, which has been associated with a number of long-term neurocognitive effects (Cacioppo et al., 2011 ). This is standard procedure in our laboratory for animals with surgical implants, as well as those maintained on food restriction, but should be minimized as much as possible.

Aside from concerns about animal welfare, an experimental factor that must be considered is the study duration. BSR studies are likely longer than studies using sugar pellets. Animal recovery from surgery and the time required for rate–frequency curve stabilization can add weeks of training before the task of interest can be completed. However, shaping animals to lever-press for BSR is typically much faster than shaping animals for sugar pellets (1–2 d vs. 1–2 wks). In our experiments, the total duration of the BSR magnitude discrimination task was ∼8–9 wks, whereas the sugar pellet magnitude discrimination task required 5–6 wks. Depending on the reward sizes used, it may also be possible to skip the rate–frequency curve step of training, decreasing the duration of BSR studies. Indeed, some success using this approach has already been demonstrated (Tedford et al., 2015), but it is unknown how this approach may impact the accuracy of results. Additionally, to accommodate for the decreased stability of pellet responding, investigators could shorten behavioral sessions; however, additional days of testing would then be required to achieve a critical number of trials. Thus, well-controlled versions of the pellet-rewarded tasks may require as long a duration as BSR-rewarded tasks. Last, the use of BSR requires a substantial amount of specialized (and expensive) equipment, which may be prohibitive to some laboratories. Despite these potential issues, BSR still presents an important opportunity for more consistent and accurate reward-motivated behavioral testing, and should be strongly considered by investigators in this field.

## Synthesis

Reviewing Editor: Daniela Schiller, Mount Sinai School of Medicine Decisions are customarily a result of the Reviewing Editor and the peer reviewers coming together and discussing their recommendations until a consensus is reached. When revisions are invited, a fact-based synthesis statement explaining their decision and outlining what is needed to prepare a revision will be listed below. The following reviewer(s) agreed to reveal their identity: Jonathan Lee.

Food is typically used as a positive reinforce for studying decision-making in rats. But there are a number of potential confounds associated with food reinforcement. Recently, electrical brain stimulation reward was shown to be an effective reinforcer for these tasks. This report provides for the first time direct comparisons between electrical brain stimulation and food as positive reinforcers for decision-making tasks in rats. There were several outstanding features of the studies; they were well-designed and executed, with excellent control comparisons. The outcomes were properly interpreted, data presentation was clear, and the paper was particularly well written. This report will aid in the interpretation of historical studies and will guide future studies on decision-making by rats.

The design of the experiments is largely appropriate to address the aims. There is a good rationale for the selection of BSR frequencies, as well as number of sucrose pellets. Care has been taken to equate the two experiments as much as possible. There are a few potential concerns, however, which the authors could addressed in a revision:

- Introduction - Page 3, lines 61 - 67 ... “we know little about the discriminability of BSR in tasks where animals freely choose between multiple rewards, within-session consistency of BSR choice....”. The next sentence continues with “Here, we compared the discriminability of BSR frequencies and sugar pellet numbers in similarly-structured free-choice tasks, where animals selected the preferred reward of two options within the same reward modality.” Basically, if reward magnitudes were discriminable, animals should select the larger of the two rewards.' The cited Rokosik and Napier, 2011 study (as well as follow up studies from the Napier lab; Rokosik and Napier, 2012; Tedford, et al., 2015; and Holtz, et al., 2016) all already demonstrated that rats can discriminate between BSR reward magnitudes, based on current frequency (as well as current magnitude) in tasks where the rats freely choose between two rewards, and showed within-session consistency of BSR choice. The novel contribution of the current study is that the authors systematically determined the relationship between the size of BSR frequency and the discrimination capacity (or acuity) of the rats. This paragraph should be rewritten to more properly represent the state of the art.

- Experimental design - It appears from the methods that the different BSR frequencies are first associated with discriminable auditory tones prior to the magnitude discrimination task, whereas there was no such tone-facilitated discrimination for the sucrose pellets. This difference is neither justified in the methods, nor addressed in the discussion. Is it not possible that this pre-training might enhance the magnitude discrimination in some way? It would be helpful to the reader to appreciate the potential importance of such pre-training. Furthermore, the experiment is not counterbalanced in that BSR magnitude discrimination is conducted before sucrose magnitude discrimination across all subjects. This should be acknowledged, as there are unknown potential effects of this upon the sucrose data. The existing discussion of the sucrose data can certainly be used to argue against a major effect, but it would be nice to have such a discussion. Clearly, neither of these issues affects the central conclusion of the study, as adding pre-training to the sucrose protocol, or conducting the sucrose discrimination first, would not reduce the critical issue of satiety and reduced task engagement.

- What was the nature of the food restriction? There is evidence, especially for decision-making studies, that the nature of food restriction is an important factor in the stability of behavior.

- The current discussion makes a compelling case for the advantages of using BSR. These are mainly the stability of reward magnitude discrimination across the course of a behavioral session and the potential utility of using very small differences in reward size. It would be helpful first to be a little more explicit how the latter would facilitate “a richer understanding of decision-making and its underlying neurocircuitry”. This comes under a broader issue, which is largely omitted from the discussion, yet is incredibly important from a forward-looking perspective. There is a clear case made for the potential benefits of using BSR, rather than sucrose. However, the field would greatly benefit from at least an introduction to a framework of issues to consider in the evaluation of BSR vs sucrose reward. Specifically, BSR undoubtedly adds a further welfare burden to the animals (due to the surgical intervention), which must be considered as a cost in comparison to food restriction and sucrose reward. The methods and results do not currently make it clear enough how comparable the two procedures are in terms of duration of training (given that pre-training appears important for BSR). This is another potential, albeit minor, cost. Part of the calculation might be the additional testing required were sucrose sessions to be shortened to a duration that is less affected by within-session decline in behavioral output. Therefore, overall, the authors might have missed an opportunity to guide the field more thoroughly in the cost-benefit analysis that should be undertaken when considering BSR vs sucrose.

Minor comments:

- the emphasis is on studies of decision-making, for which long sessions with many trials are traditionally employed. Perhaps this latter point might be emphasized a bit more explicitly, especially for the benefit of those readers who might approach the published paper from a non-decision-making background. This is especially important when the data on reduced task engagement in the latter portions of sucrose sessions is considered.

- it would help to state the total number of rats used at the very beginning of the methods.

- there is an exclusion criterion stated for the BSR data. It should be made clear how many rats were excluded on this basis. This is another important element in the risk-benefit analysis mentioned above.

- page 2, lines 50-51 states “may have unforeseen behavioral consequences ...”. This is ambiguous. Providing examples would be helpful.

- Page 3. Line 77 states that the rats were single housed. This is a known stressor to rats (who normally live in groups) that can alter cognitive outcomes. The authors should acknowledge this.

- Page 4, Line 93. Maloxicam is a trade name - please indicate as such and/or provide the generic.

- Page 4, Lines 96-97 state that “only subjects with accurate electrode placements ...”. How was electrode placement determined? Was placement visualized post mortem?

- Page 7, lines 221 and 223. Correct “posthoc” to read post hoc, and “tukey” to read “Tukey”.

- Page 8, line 225 states that “All graphical results are presented as means {plus minus} sem”. However, the bar graphs only show means + sem.

- Page 12, 383-384 states that, “these results suggest that BSR may be an effective reinforcer in rodent decision-making tasks”; when in fact the results provided support for prior reports (Napier and Rokosik 2011, 2012; Tedford et al 2015; and Holtz et al. 2016), which have already determined that BSR is an effective reinforcer in decision-making tasks.
